# Computed Tomography Dose Level in Selected Five Principal Hospitals in Ethiopia

**DOI:** 10.4314/ejhs.v33i6.11

**Published:** 2023-11

**Authors:** Gebremedhin Kide Kinfe, Birhanu Tsegaye Wores

**Affiliations:** 1 Department of Physics, College of Natural and Computational Sciences, Aksum University

**Keywords:** Effective Dose, Ionizing Radiation, Dose Level, Ethiopia

## Abstract

**Background:**

X-ray Computed Tomography dose levels have been varying among modalities and scanning body regions due to the absence of incessant routine follow-up. Thus, the study aimed to compute the dose index discrepancies in Ethiopia for the most recurring scan protocols (head, chest, abdomen, and pelvis).

**Methods:**

A purposive sampling method was employed to select the hospitals due to the rare existence of functional CT scanners in Ethiopia. From the selected hospitals, a total of 1,385 (249 heads, 804 chests, 132 abdomens, and 200 pelvis) were collected in terms of standard dose metric values in the period of December 2019-March 2020. Patients' DLP was computed into mean value using IBM SPSS Statistics 20 software. From the mean DLP, we can compute the effective dose.

**Results:**

Patients' dose level disparity was observed in this study though it is below the ICRP standard level for all body regions except for pelvis DLP (593.37 mGy-cm) at Black Lion. The dose level for the head and chest are computed within the recommended level at all hospitals. Effective doses for the pelvis at four hospitals (Teklehaimanot, Black Lion, ALERT, Paul's, and Ayder hospitals) were computed as 6.45, 8.90, 5.08, 6.54, and 6.84 mSv respectively, and the effective doses for abdomen at Ayder Hospital was obtained to be 8.90 mSv, which is above the recommended value.

**Conclusion:**

X-ray CT scanners are somewhat properly functioning although some sort of justification and optimization for pelvis and abdomen examinations are strongly recommended to implement as low as reasonably achievable principle.

## Introduction

X-ray Computed Tomography (X-ray CT) is a computerized diagnostic imaging technique applied in the diagnosis, treatment planning, and localization of tumors, bone fractures, and other physiological activities of human organs without any sort of surgical operation in a safe way (1). However, the International Commission on Radiological Protection (ICRP), the International Atomic Energy Agency (IAEA), and the European Union of Radiation Protection advocated that X-ray CT is associated with a high dose, which level of measurement needs to be optimized to carry out clinically needed radiation exposure levels. This can be achieved through continuous dose and performance follow-up. The measurement of patient dose in diagnostic imaging has been earlier than half a century (2).

Moreover, the Biological Effects of Ionizing Radiation Committee (BEIR) of the American National Academy of Sciences provides a useful guide on radiological risks in patients. As reported in the guide, higher-level radiation dose exposure induces stochastic hazards that bring up to spread of cell killing. Even continual and gradual exposure to small doses can cause possible biological risks, as there is no risk-free radiation. Such kind of approach is the “linear non-threshold” model (3). Thus, it is critically significant to measure the X-ray CT dose level and regular follow-up through medical specialists (medical physics, nuclear medicine, radiotherapy, and radiology) (4).

In the Ethiopian context, imaging modalities performance follow-up through regular dose level analysis is insufficient except that there are very few studies that have been carried out by a few researchers who have studied radiation exposure awareness and collective dose distribution of the human body (5, 6). In Ethiopia, there are no clear standard guidelines and regulations on ionizing radiation utilization and protection protocols. These may cause a serious biological detriment to patients subjected to imaging procedures and low customer confidence in the care they receive from diagnostic radiologists. It is, therefore, the biological detriment of ionizing radiation, the necessity to compute the exposure level conveyed from X-ray CT machines to the patients.

The result obtained in this study is critically essential for radiation protection and optimization through diagnostic reference level establishment. Taking into consideration such recommendations, the present work has been able to record the CTDI_vol_ (mGy) and DLP (mGy.cm) and estimate the effective dose (ED) based on the dose index and the conversion factors. The mean values of reported CTDI_vol_, DLP, and computed effective dose were calculated for each protocol (head, chest, abdomen, and pelvis) and were compared among the selected hospitals, Internationally reported values, and with ICRP standards.

Computed tomography volume Dose Index (CTDI_vol_) is a standardized method to measure radiation output from the scanner. These dose measurements are obtained from 16 or 32-cm diameter acrylic phantoms (16 cm head CT and 32 cm body CT). The phantom size used for its calculation is displayed on CT and measured in milligrams (mGy) (11). CTDI_vol_ corresponds to the average absorbed radiation dose or intensity of radiation exposure per slice over the x, y, and z-axis of the patient being examined. It details the overlaps or gaps between the ionizing radiation beams from successive rotations of the X-ray tube (12,13). It is a useful indicator of radiation dose for a specific examination because it represents the average dose within each slice (14). The drawback of this dosimetry is that it does not represent actual patient doses (the dose for patients of different sizes, shapes, and attenuation), and it does not indicate the total energy deposited into the scan volume because it is independent of the total scan length (15).

Dose length product is another dosimetric quantity collected from the scanner dose-reporting page. It points out the distribution of radiation exposure in the course of scanning (16). DLP does not represent patient-specific doses. It is the product of the intensity of ionizing radiation exposure level (denoted by the CTDI_vol_) and the distribution (denoted by the length of the patient's scanned anatomy) (17,18). Mathematically it is defined as:


$$\text{DLP}={\text{CTDI}}_{\text{vol}}{\;}^{*}\;L\;\ldots \ldots \ldots \ldots \ldots \ldots \ldots (1)$$

DLP is directly related to scan length, i.e. as the scan length increases it also increases and vice versa, while CTDI_vol_ remains unchanged with scan length. During the examination, DLP is higher for taller patients' anatomy and smaller for shorter ones. For that reason, DLP is carefully selected in this study for determination of the dose level among patients of different sizes, shapes, and attenuation since it is a symbol of both the intensity of radiation and scan length, while CTDI_vol_ represents only the intensity of the radiation (19-21).

After the mean value of DLP was calculated for each of the anatomic body regions at five selected hospitals using IBM SPSS statistics 20 software, the effective dose (ED) stated in equation (2) was obtained by multiplying the mean DLP value and the tissue normalization constant (E_DLP_) based on the report from (22-24):


$$E={\text{DLP}}^{*}{\;E}_{\text{DLP}}\ldots \ldots \ldots \ldots \ldots \ldots \ldots \;(2)$$

Where ED (mSv) is the effective dose, DLP (mGy-cm) is the dose length product and E_DLP_ (mSv.mGy^-1^cm^-1^) is the tissue normalization constant as defined separately for various scan ranges in Table 1.

**Table 1:** Descriptive statistics of DLP (mGy-cm) at five X-ray CT scanning centers of the anatomic body region

Anatomic Body Regions	Range	Minimum	Maximum	Mean	Mean of Std. Error	Std.
Head	184.40	760.20	944.60	893.58	34.14	76.34
Chest	82.40	296.20	378.60	325.76	14.48	32.39
Abdomen	248.40	345.00	593.40	438.70	41.27	92.28
Pelvis	254.70	338.70	593.40	450.80	40.97	91.62

Finally, the findings can guide medical experts working on radiation-related activities to optimize ionizing radiation into desired and accepted dose levels that enable the reduction of cancer and other related possible biological risks of patients who are exposed to X-ray CT diagnostic imaging in Ethiopia.

## Materials and Methods

In this study, a purposive method of sampling was employed to select the five hospitals, as there are a limited number of hospitals having CT scanners that display standardized dose metrics (CTDI_vol_ and DLP) and other dosimetric parameters on the computers' control console. Based on this sampling, we were able to get one hospital from Mekelle City in Tigray and four hospitals from Addis Ababa, which is a metropolitan city in Ethiopia. From the selected hospitals, a total of 1,385 (249 heads, 804 chests, 132 abdomens, and 200 pelvis) were collected in terms of standard dose metric values in the period of December 2019-March 2020.

Before commencing the data collection process, a permission request letter attached with a proposal and the ethical clearance certificate offered by Aksum University College of Health Science was submitted to every hospital. As we got an official permit from all five hospitals, the questionnaires prepared were distributed among radiologists and patients. Questionnaires for radiologists tried to answer modalities model, standardized dose metrics, and other diametric parameters display and for patients to provide us with weight and the existence of gross pathology or not.

As per the prepared questionnaire and the dosimetric parameters displayed on the computer control console, sufficient data were collected in terms of CTDI_vol_, and DLP for anatomic body regions: brain, chest, abdomen, and pelvis based on the recommendation of (7). In so doing, the machines used were Neu Viz (Teklehaimanot General Hospital), Optima™ CT660 (Black Lion Specialized Hospital), Brilliance (The All Africans Leprosy, Tuberculosis, and Rehabilitation Training Center (ALERT) General Hospital), Optima™ CT660 (St. Paul's Millennium Medical College), and Bright Speed (Ayder Specialized Hospital); 64-slice image acquisition modeled X-ray CT scanners manufactured by GE healthcare.

Furthermore, data with complete patient information such as age, examination date, dose indexes, and report descriptions were recorded by refining less commonly performed patient information such as gross pathology, contrast-enhanced procedures, repeated imaging procedures, and mixed protocols to retain from over-dose estimation (8). Medium-sized patients weighing 50-90kg were also included based on the information in (9) by making patient interviews because the modality's patient dose information software could not display the patient size.

**X-ray CT dose quantities:** X-ray Computed Tomography standardized dose metrics (CTDI_vol_ and DLP) were documented for detailed analysis. Based on CTDI_vol_ and DLP, values were obtained liable for the comparison of dose-measured exposure levels which are universally interpreted in risk management (10).

The data collected from the five hospitals were analyzed using IBM SPSS Statistics 20 software to quantify dosimetric quantitative variables such as mean, and mean std. Error, range, standard deviation of DLP, and ED for each examination according to the guideline in (25-27). The mean of DLPs in this study was calculated for each anatomic region and a comparison was made among hospitals revealing statistically significant differences between the dose levels. In addition, graphs were prepared using OriginPro 8 software.

Before the data collection was done, an ethical clearance permit was collected from Aksum University, the College of Health Sciences Referral Hospital research, and the ethical clearance board. According to the health-related ethical clearance rule, the information gained from the direct rooting diagnostic imaging procedures would not be used outside the scope of the study. Eventually, any information that was obtained during the study would be kept confidential.

## Results

A total of 1,385 (249 heads, 804 chests, 132 abdomens, and 200 pelvis) absorbed dose (CTDI_vol_) and distributed dose (DLP) values displayed on the computer control console were collected from the five hospitals in Ethiopia. The descriptive statistics of the DLP for the four anatomic body regions scanned in different hospitals are reported as displayed in Table 1.

As illustrated in Table 1, significant discrepancies in DLP levels were observed among patients, which can be mainly seen due to the nature of the tissue density to absorb ionizing radiation and technical variations such as parameter adjustment and phase scanning employed (1). The highest mean standard deviation with 92.28 mGycm and 91.62 mGycm for the abdomen and pelvis respectively was recorded whereas the mean standard errors for all body regions showed significant differences because a smaller number of patients were subjected to the scanning procedures (8).

The effective doses computed for each anatomic body region obtained from the product of DLP and tissue normalization (K) as recommended by the ICRP for the five different hospitals are tabulated in Table 2. The result indicated that some significant discrepancies in effective dose (mSv.) were seen among different hospitals for the same anatomic body region mainly caused by the variation in DLP levels. For instance, the highest ED for the head with 2.0 mSv is recorded at Black Lion and Ayder hospitals whereas the lowest one with 1.6 mSv at Tecklehaimanot; for the chest the highest one with 4.49mSv is obtained at St. Pual's whereas the lowest one is Black Lion; for abdomen, the highest one with 8.90 mSv is reported at Ayder whereas the lowest one with 5.18mSv is at Teklehaimanot; and finally, for the pelvis, the highest one with 8.90 mSv was obtained at Black Lion whereas the lowest one was 5.08mSv at ALERT.

**Table 2:** Effective Dose(mSv) computed from the mean DLP and the tissue normalization constant K(mSvmGy^-1^-cm^-1^)for body regions head, chest, abdomen, and pelvis at each hospital

Hospital's Name	Anatomic Body Regions			

	Head	Chest	Abdomen	Pulvis
Teklehaimanot	1.6	4.52	5.18	6.45
Black Lion	2.0	4.15	6.38	8.90
ALERT	1.9	5.30	6.30	5.08
St Pual's	1.9	4.59	6.16	6.54
Ayder	2.0	4.24	8.90	6.84

The dose levels of DLP and ED computed in the current study for the considered anatomic body regions have been compared and analyzed following the recommended optimal dose levels by the International Commission on Radiation Protection (ICTP) as illustrated in Table 3.

**Table 3:** ICRP Suggested effective dose, DLPs, and Tissue Normalization Constant [27, 28, 29] for body regions (head, chest, abdomen, and pelvis)

Anatomic Body Region	ED (mSv)	DLP (mGy-cm)	K (mSv mGy^-1^-cm^-1^)
Head	1-2	1050	0.0021
Chest	5-7	650	0.014
Abdomen	5-7	780	0.015
Pelvis	3-4	570	0.015

## Discussion

Nowadays, the measurement of dose exposure levels in patients has become a common practice in the world due to the increasing X-ray CT medical imaging and the induced collective dose on the public. In this regard, patient dose level analysis in selected five principal hospitals in Ethiopia has been conducted for the recurring anatomic body regions. The selection of anatomic body regions is based on the ICRP recommendations for the recurrent imaging procedures as to the report in (28).

The dose recorded from the CT computer control console was compared with standards as suggested by the ICRP and the internationally reported values to observe dose discrepancy among modalities and assess the modality performance. This result is analogous to other reports (28, 29). The type of scanner and protocols used also induce dose variations in patients which is in line with the report of guidance on the establishment and use of diagnostic reference levels for medical X-ray examinations (16).

**Figure 1:**
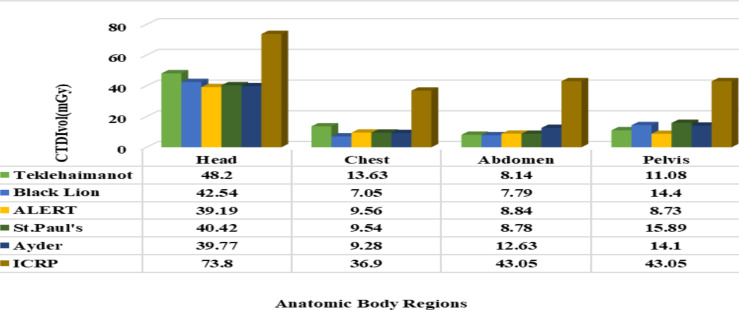
Graphical comparison of CTDI_vol_ among the selected hospitals and with ICRP recommended dose level

**Figure 2:**
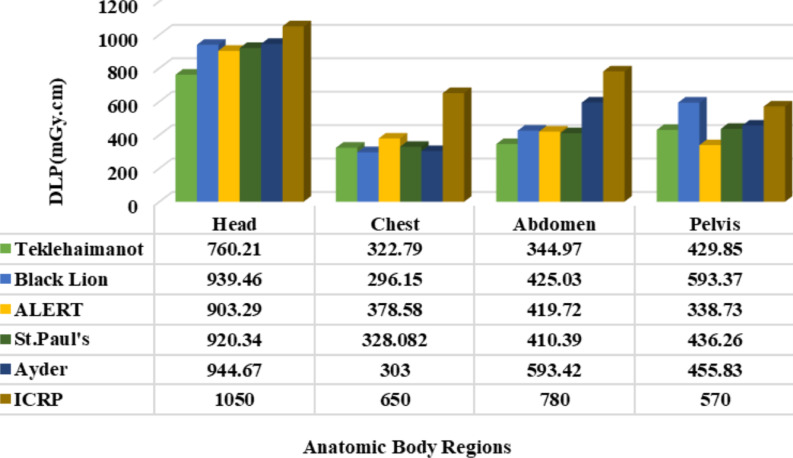
Graphical comparison of DLP among the chosen hospitals and with ICRP recommended dose level

In all the hospitals considered in this study, the recorded level of CTDI_vol_ was below the international recommended dose level as depicted in Figure 1, which is the best exposure level as to the ICRP recommendation. A similar study was reported (30), whereas the result attained in the current study was the best as compared with a related study carried out elsewhere (31,32), which is above the standard for head imaging. Concerning the Dose Length Product (DLP), the individual patients' dose length product was computed to mean values for comparative analysis among hospitals as illustrated in Figure 2. Accordingly, the DLP record was found a little higher than the ICRP standard for pelvis scanning (593.37 mGy.cm) at Black Lion due to the maximum scan length used. However, at all other hospitals, the DLP values were found within the appropriate recommended limit. Moreover, the relationship between scan length with DLP was identified shows reasonably high DLP in large body-sized patients (large BMI, and weight) patients, and large scan length CT scanning. In the current study, the average BMI (19.8 kg/m^2^) and weight (58 kg) was computed. Individual patient's body size and the dose absorbed show a direct proportion. This is because the X-ray beam attenuates principally in large-sized patients due to the many scattering events presented in the ICRP publication 103. Ann. ICRP 2007 (33). The result of this study is better than the similar studies reported by (34-36).

Regarding the effective dose (ED), it is an indicator of biological sensitivity to ionizing radiation among patients used to compare doses from different diagnostic imaging procedures. Moreover, ED is used for comparing the use of similar X-ray CT technologies and procedures in different centers as well as the use of different technologies for the same diagnostic imaging procedure (37).

**Figure 3:**
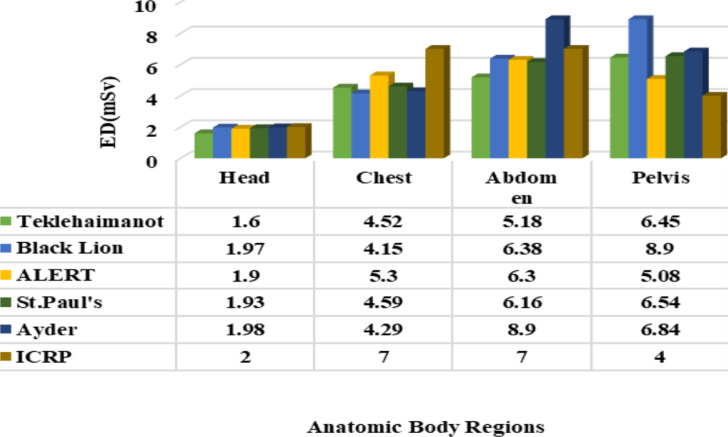
Graphical comparison of effective dose among the target hospitals and with ICRP recommended dose level

According to the CT dosimeter comparison of measurement techniques and devices, an effective dose below the allowed level has an infrequent biological effect (35), whereas above the suggested level may lead to tissue cell damage and or be modified to develop into cancer after a prolonged delay as presented in a study (38). In line with this principle, the result achieved during head and chest examination is below the ICRP recommended level which is similar to the reports in other studies (30, 32). In addition, an ED below the recommended level was computed for the abdomen at all hospitals except at Ayder (8.9mSv), which is above the standard due to the large DLP record as shown in Figure 3. However, an ED beyond the internationally suggested level was computed for the pelvis in the five hospitals considered in this study due to the large DLP and scan length made. The increase in scan length of these parameters leads to an increase in DLP and then to ED in a direct proportion. As DLP increases, the effective dose also increases for constant values of E_DLP_ (25). For instance, the highest DLP distribution recorded at Black lion (593.4mGy.cm) triggers a higher level of ED (8.90 mSv.), while the smallest one at ALERT (338.70 mGy.cm) leads to the smallest ED (5.08 mSv) for pelvis scanning. Even though the current result is higher than the standard for the pelvis, it is somewhat better than other findings reported (22, 36, and 39).

In summary, the result shows that the CTDI_vol_, DLP, and ED for the brain and chest were measured significantly lower compared to the ICRP standard except for the pelvis, which was recorded above the standard. Similarly, the effective dose during abdomen diagnostics in Ayder Hospital and DLP during pelvis examination at Black Lion was above the international standard. As well, dosimetric parameters vary in the centers.

Based on the result of this study, the researchers recommend the following points:

- The necessity of X-ray CT dose level measurement especially for pelvis and abdomen diagnostic imaging needs further dose optimization for optimal diagnostic imaging quality and performance.
- Optimization of the X-ray CT doses based on BMI and patient size is entailed.

Further large-scale dose inspection in all diagnostic and nuclear medicine facilities across the nation is required, and the current data can be used to establish clinical diagnostic reference levels as an aid for dose optimization in Ethiopia.

## References

[1] Mettler FA, Huda W, Yoshizumi TT, Mahesh M (2008). Effective doses in radiology and diagnostic nuclear medicine: a catalog. Radiology.

[2] Vañó E, Miller D, Martin C, Rehani M, Kang K, Rosenstein M, Ortiz-López P, Mattsson S, Padovani R, Rogers A (2017). ICRP publication 135: diagnostic reference levels in medical imaging. Annals of the ICRP.

[3] De González AB, Kim KP, Knudsen AB, Lansdorp-Vogelaar I, Rutter CM, Smith-Bindman R, Yee J, Kuntz KM, Van Ballegooijen M, Zauber AG (2011). Radiation-related cancer risks from CT colonography screening: a risk-benefit analysis. AJR American journal of roentgenology.

[4] Gao Y, Quinn B, Mahmood U, Long D, Erdi Y, St Germain J, Pandit-Taskar N, Xu XG, Bolch WE, Dauer LT (2017). A comparison of pediatric and adult CT organ dose estimation methods. BMC Medical Imaging.

[5] Zewdneh D, Dellie ST, Ayele T (2012). A study of knowledge & awareness of medical doctors towards radiation exposure risk at Tikur Anbessa specialized referral and teaching hospital, Addis Ababa, Ethiopia. J Pharm Biol Sci.

[6] Asefa G, Getnet W, Tewelde T (2016). Knowledge about radiation-related health hazards and protective measures among patients waiting for radiologic imaging in Jimma University Hospital, Southwest Ethiopia. Ethiopian Journal of Health Sciences.

[7] Qu X-m, Li G, Ludlow JB, Zhang Z-y, Ma X-c (2010). Effective radiation dose of ProMax 3D cone-beam computerized tomography scanner with different dental protocols. Oral Surgery, Oral Medicine, Oral Pathology, Oral Radiology, and Endodontology.

[8] Bolowia N (2018). Establishment of computed tomography diagnostic reference levels in Tobruk. J Med Diagn Meth.

[9] Morin RL, Gerber TC, McCollough CH (2003). Radiation dose in computed tomography of the heart. Circulation.

[10] McNitt-Gray MF (2002). AAPM/RSNA physics tutorial for residents: topics in CT: radiation dose in CT. Radiographics.

[11] Kadavigere R, Sukumar S (2022). Estimation of radiation dose and establishment of local diagnostic reference levels for computed tomography of head in the pediatric population. Journal of X-Ray Science and Technology.

[12] Kanal KM, Butler PF, Sengupta D, Bhargavan-Chatfield M, Coombs LP, Morin RL (2017). US diagnostic reference levels and achievable doses for 10 adult CT examinations. Radiology.

[13] Simantirakis G, Hourdakis C, Economides S, Kaisas I, Kalathaki M, Koukorava C, Manousaridis G, Pafilis C, Tritakis P, Vogiatzi S (2015). Diagnostic reference levels and patient doses in computed tomography examinations in Greece. Radiation protection dosimetry.

[14] Chinem LAS, Vilella BdS, Maurício CLdP, Canevaro LV, Deluiz LF, Vilella OdV (2016). Digital orthodontic radiographic set versus cone-beam computed tomography: an evaluation of the effective dose. Dental Press Journal of Orthodontics.

[15] Protection ICoR (2001). Diagnostic reference levels in medical imaging: review and additional advice. Ann ICRP.

[16] IPEM (2004). Guidance on the establishment and use of diagnostic reference levels for medical X-ray examinations. Institute of Physics and Engineering in Medicine DRL Working Party Report 88.

[17] Vassileva J, Rehani M (2015). Diagnostic reference levels. AJR Am J Roentgenol.

[18] McCollough CH (2010). Diagnostic reference levels. Image Wisely [Internet].

[19] McNitt-Gray MF (2002). AAPM/RSNA physics tutorial for residents: topics in CT: radiation dose in CT. Radiographics.

[20] McCollough CH, Leng S, Yu L, Cody DD, Boone JM, McNitt-Gray MF (2011). CT dose index and patient dose: they are not the same thing. Radiology.

[21] Huda W, Mettler FA (2011). Volume CT dose index and dose-length product displayed during CT: what good are they?. Radiology.

[22] Huda W, Mettler FA (2011). Volume CT dose index and dose-length product displayed during CT: what good are they?. Radiology.

[23] Monti LD, Setola E, Fragasso G, Camisasca RP, Lucotti P, Galluccio E, Origgi A, Margonato A, Piatti P (2006). Metabolic and endothelial effects of trimetazidine on forearm skeletal muscle in patients with type 2 diabetes and ischemic cardiomyopathy. American Journal of Physiology-Endocrinology and Metabolism.

[24] Laurier D, Ruehm W, Paquet F, Applegate K, Cool D, Clement C, Protection ICoR (2021). Areas of research to support the system of radiological protection. Radiation and Environmental Biophysics.

[25] Charles MW (2008). ICRP Publication 103: Recommendations of the ICRP.

[26] McCollough C, Cody D, Edyvean S, Geise R, Gould B, Keat N, Huda W, Judy P, Kalender W, McNitt-Gray M (2008). The measurement, reporting, and management of radiation dose in CT. Report of AAPM Task Group.

[27] Goldman LW (2007). Principles of CT: radiation dose and image quality. Journal of Nuclear Medicine Technology.

[28] Shrimpton PC, Jansen JT, Harrison JD (2016). Updated estimates of typical effective doses for common CT examinations in the UK following the 2011 national review. The British Journal of Radiology.

[29] Valentin J (2005). International Commission on Radiological Protection. Biological effects after prenatal irradiation (embryo and fetus).

[30] Maharjan S, Prajapati S, Panta O (2016). B. Measurement of radiation dose in multi-slice computed tomography. Bangabandhu Sheikh Mujib Medical University Journal.

[31] Foley S J, McEntee M F, Rainford L A (2012). Establishment of CT diagnostic reference levels in Ireland. The British Journal of Radiology.

[32] Suliman I, Abdalla S, Ahmed N A, Galal M, Salih I (2011). Survey of computed tomography technique and radiation dose in Sudanese hospitals. European Journal of Radiology.

[33] International Commission on Radiological Protection (2007). ICRP publication 103. Ann. ICRP.

[34] Razali M, Ahmad M, Roslee M, Osman N (2019). Establishment of an institutional diagnostic reference level for CT imaging associated with multiple anatomical regions. Paper presented in the Journal of Physics.

[35] Ekpo E U, Adejoh T, Akwo J D, Emeka O C, Modu A A, Abba M, Chiegwu U H (2018). Diagnostic reference levels for common computed tomography (CT) examinations: results from the first Nigerian nationwide dose survey. Journal of Radiological Protection.

[36] Smith-Bindman R, Moghadassi M, Wilson N, Nelson T R, Boone J M, Cagnon C H, Lamba R (2015). Radiation doses in consecutive CT examinations from five University of California Medical Centers. Radiology.

[37] Shirazu I, Mensah Y, Schandorf C, Mensah S Estimate of Reference Effective Dose and Renal Dose during Abdominal CT Scan for Dose Optimization Procedures in Ghana.

[38] Podgorsak E (2008). Radiation oncology physics: a handbook for teachers and students. British Journal of Cancer.

[39] Ramzee S, Surujpaul P, Sayan C (2019). Patient Dose Audit in Computed Tomography at Cancer Institute of Guyana. J Med Diagn Meth.

